# Pet Ownership and Children’s Emotional Expression: Propensity Score-Matched Analysis of Longitudinal Data from Japan

**DOI:** 10.3390/ijerph16050758

**Published:** 2019-03-02

**Authors:** Rikako Sato, Takeo Fujiwara, Shiho Kino, Nobutoshi Nawa, Ichiro Kawachi

**Affiliations:** 1 Department of Global Health Promotion, Tokyo Medical and Dental University, Tokyo 113-8519, Japan; 150502ms@tmd.ac.jp (R.S.); nobujpjp@gmail.com (N.N.); ikawachi@hsph.harvard.edu (I.K.); 2 Department of Social and Behavioral Sciences, Harvard T.H. Chan School of Public Health, Boston 02115, MA, USA; shkino@hsph.harvard.edu

**Keywords:** Pet ownership, child development, emotional regulation, Japan, propensity score matching

## Abstract

With many children and young adolescents reporting strong emotional bonds with their pets, the impact of pet ownership on child/adolescent health—especially on their emotional development—has garnered increasing scientific interest. We examined the association between pet ownership in toddlerhood (age 3.5 years) and poor emotional expression in later childhood (age 5.5 years) using propensity score matching within a longitudinal cohort dataset from Japan (*n* = 31,453). A propensity score for pet ownership was calculated by logistic models based on a comprehensive list of each child’s observed characteristics, including sex, household income, parental education, mother’s employment status, residential environment, number of siblings, and living arrangement. Log-binomial regression analyses using matched samples revealed that children who owned pets during the toddler years were 6% less likely to have a poor emotional expression in later childhood (prevalence ratio = 0.94, 95% confidence interval = 0.90–0.99) compared to those without pets. This suggests that owning pets may provide children with opportunities to control their emotions, and lead to a lower prevalence of poor emotional expression. Pet ownership in toddlerhood may contribute to the development of expression.

## 1. Introduction

Companion animals are a key element of life for many people. In the U.S., 68% of all households own one or more pets [1], and similar rates of ownership are found across Europe, Australia, China, and Japan [2]. A variety of health benefits have been claimed for pet ownership in adults, including lower risk of cardiovascular disease [3,4] lower blood pressure, increased physical activity, and increased social interactions with other people [5,6,7,8,9].

While most of these studies have focused on midlife and older adults, there is increasing interest in examining the effects of pet ownership among children, such as less allergic manifestations [10], lower prevalence of hypertension [11]. Early childhood is a critical phase in the life course when interactions with others can shape the course of child development and emotional wellbeing [12,13,14,15]. Indeed, some studies have reported an association between pet ownership and children’s emotional development, including the development of empathy, emotion regulation, enhancement of self-esteem and reducing feelings of loneliness; however, the findings stemmed mainly from examining adolescent samples [16,17,18,19,20,21,22].

To the best of our knowledge, few studies have investigated the association between pet ownership and emotional expression among young children, such as preschool children, have been published. Preschool age is an important stage of communication development [12], and emotional expressiveness is a key aspect of good communication skills [23]. Since it is reported that pet ownership is associated with a higher social function, including communication skills for older people [24], we hypothesized that pet ownership may have a similar impact on preschool children by stimulating their emotional expressiveness. 

To date, the majority of studies on pet ownership and children’s emotional health have been either cross-sectional, or they failed to adequately take account of background confounding factors [25]. For example, parents whose children are emotionally stable may afford more time to look after a pet, therefore are likely to have a pet. If so, a cross-sectional study will tend to show that children who own pets tend to be more empathic. Additionally, there may be other background differences between households that own pets versus those that do not. Pet owning households may have higher incomes, more living space, or less crowding, which may simultaneously influence a child’s emotional development, and hence confound the association between pet ownership and child developmental outcomes. An ideal strategy would be to conduct a randomized trial of pet ownership, but this is difficult for both practical and ethical reasons. 

Hence, we must adopt an analytic strategy that mimics the randomized trial by matching pet owners and non-owners on their propensity to own a pet. Here, we compare the emotional expression between children in households that are as alike as possible (exchangeable with each other), except for the fact that one group happens to own a pet, while the other does not. Accordingly, we conducted a propensity score-matched analysis of pet ownership and children’s emotional expression within a longitudinal dataset from Japan.

## 2. Materials and Methods 

### 2.1. Data and Samples

The data were obtained from the Longitudinal Survey of Newborns in the 21st Century, a population-based cohort data set collected by the Japanese Ministry of Health, Labor, and Welfare. The study sample included all newborns born between 10 and 17 January, 2001, or 10 and 17 July, 2001, in Japan, using the birth records from Japan’s national vital statistics (*n* = 53,575). The baseline survey was mailed to parents when their infants were six months old. Since then, annual surveys have been mailed to participants.

We used data from the 2001, 2004, and 2006 survey waves. A total of 47,015 caregivers responded to the baseline questionnaire (response rate: 87.7%). Of these, 37,937 caregivers (88.4%) responded to the 2004 survey wave (when the children were 3.5 years old). The response to the 2006 surveys (when the children were 5.5 years old) was 82.0%. We excluded the individuals with missing data for the variables used in the analyses. The missing samples were similar with samples without missing data for child sex (boy: 51.9%) and pet ownership (37.3%). However, household income (first quartile: 33.0%) and parental education (mother graduated high school (HS): 60.8%, father graduated HS: 55.4%) were lower than in samples without missing data. In total, 31,453 newborns were included (66.9% of the total respondents) in the analyses.

### 2.2. Variables

*Pet Ownership.* Data on pet ownership of the household were retrieved from data collected in the 2004 survey wave, when the children were 3.5 years old. On the wave, caregivers were asked if the household owned a pet and if so, what kind of pet (dog, cat, rabbit, small animals (such as hamster), bird, insect, fish, reptile, or others). In our analysis, we categorized the study population into two groups (pet owners and non-owners).

### 2.3. Outcome

Emotional expressiveness was assessed through self-report by the caregivers when the children were 5.5 years old (on the 2006 survey wave). Here, caregivers were asked the following question: “Does your child have difficulty in expressing their emotions well?” with a “yes/no” response. This item was previously used to assess the behavioral problem in past studies [26,27] and reflects the child’s emotional expressiveness. A binary questionnaire has been used as a measurement for emotional expression in the Minnesota Preschool Affect Checklist (MPAC), a measurement of young children’s social-emotional competencies, including emotional expressiveness [28]. In this questionnaire, the observer answers whether or not the child expresses positive or negative affection, to examine their expressiveness for positive or negative affection. Our question is consistent with this questionnaire.

### 2.4. Covariates

Potential confounding factors, which could be associated with both emotional expression and pet ownership, included child sex and parental education (from the first panel study), annual household income, maternal employment status, the number of siblings, residential environment, and whether both parents were living together (from the 2004 survey wave).

The parental educational level was categorized into four groups: Lower than high school degree, high school degree, some college, and college degree or more. The annual household income was first divided by the number of people in the household to equivalize for household size. Then, the equivalized income was categorized into quartiles. The maternal employment status was categorized into five groups: Not-employed, full-time worker, part-time worker, self-employed, and other, including being a student. The number of siblings was categorized as 0, 1, and 2 or more. The residential environment was categorized into five types: Those who answered that their neighborhood was “predominantly residential”, “surrounded by factories”, “surrounded by retail stores”, “rural”, and “other”. As for the living arrangement, “not living with both parents” included children in single-parent homes, or homes where one parent was absent (due to work) for more than three months, and came back less than once in three months.

### 2.5. Statistical Analysis

All statistical analyses were conducted using Stata version 15.1 (StataCorp LLC, College Station, TX, USA).

Since the poor emotional expression was a common outcome in the sample (25%), odds ratios (ORs) will violate the rare disease assumption, and tend to overestimate the true risk ratio. Here, the log-binomial regression was conducted for each model to estimate the prevalence ratio (PR) and 95% confidence intervals (CIs) for the prevalence of children with poor emotional expression according to whether the children owned a pet or not. First, we performed the analysis in a univariate model. Then, a multivariate model was conducted, adjusting for potential confounders.

Next, propensity score matching was used to examine the relationship between pet ownership and emotional expression after accounting for other individual traits. Propensity score matching seeks to balance covariates across comparison group (owning a pet versus not-owning a pet) by matching individuals on their probability (propensity) to own a pet. To calculate the propensity scores, we selected 7 covariates, including: Child sex, annual household income, parental education, maternal employment status, residential environment, number of siblings, and whether both parents were living together. Then each child from the “owning a pet” group was matched with a child from the “not-owning a pet” group within a caliper width (the maximum permitted difference in propensity score between pairs) of 0.01 or less, while the unmatched samples were discarded. Using the matched samples, we examined the association between pet ownership in toddlerhood (3.5 years old) and poor emotional expression in later childhood (5.5 years old).

## 3. Results

The original sample included 16,345 boys and 15,108 girls. The proportion of pet owners in total, dog owners, cat owners, and owners of other kinds of pets are shown in Table 1. Of the 31,453 participants, 36.0% owned a pet in the 2004 survey wave, when they were 3.5 years old. The prevalence of pet ownership was 38.0% for boys and 33.9% for girls. Owning any kind of pet was more common among boys, however, the prevalence of dog and cat ownership did not differ by gender.

Table 2 shows the socio-demographic characteristics of respondents according to whether they owned a pet or not. In general, children owning a pet were more likely to have more siblings, less household income, less educated fathers, and employed mothers. In total, 23.0% of the children were evaluated as having poor emotional expression. The prevalence of poor emotional expression was 25.4% among boys, and 20.3% among girls.

**Figure 1:**
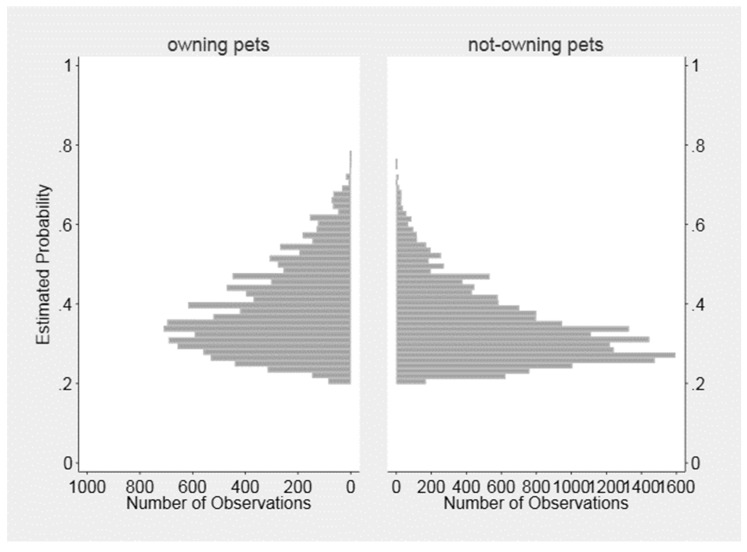
The distribution of propensity score among owning a pet vs. not-owning a pet group.

Figure 1 presents the distribution of the propensity scores according to exposure status (owning a pet vs. not-owning a pet). The histogram indicates that there is an acceptable overlap between two sets of propensity scores. The percent bias reduction of each of the individual characteristics before and after matching are reported in Table 3, which provides validation that the covariates are balanced after propensity score matching.

Table 4 presents the results of the log-binomial regression model before propensity score matching and for propensity score-matched groups. Before matching, the PR for emotional expression among pet owners in the univariate analysis was 0.94 (95% CI 0.90–0.98), and remained significant after controlling for other covariates (PR = 0.94, 95% CI 0.90–0.99). The application of propensity score matching also showed a significant result; the children owning a pet were 6% less likely to have a poor emotional expression (PR = 0.94, 95% CI 0.90–0.99).

The results of the analyses broken down by type of pet (dog, cat, or other kinds of pets) are shown in Table 5. The point estimates of the prevalence ratios of children with poor emotional expression were in a protective direction, though not statistically significant for children owning dogs (PR = 0.97, 95% CI 0.86–1.09), but significant for children owning other kinds of pet (rabbit, small animals—such as a hamster—bird, insect, fish, reptile, or others) (PR = 0.89, 95% CI 0.82–0.95), and for cat ownership, the direction was the opposite (PR = 1.08, 95% CI 0.90–1.28). The interaction between pet ownership by sex was not statistically significant; *p* = 0.605 for dog owners, *p* = 0.962 for cat owners, and *p* = 0.961 for owners of other kinds of pet.

## 4. Discussion

In this study, we examined the relationship between pet ownership and emotional expression in later childhood using propensity score matching with longitudinal data. We found that pet ownership had a modest effect on children’s emotional expressiveness, and children who had a pet at home in toddlerhood had a lower prevalence of poor emotional expression in later childhood, compared to those without pets.

Previous studies reported that people with poor emotional expressiveness are likely to feel fear against negative consequences of expressing their emotions [29]. As emotional expression is a key aspect of communication, children should acquire the ability to convey their emotions to others in a way that is advantageous for both the children and others. For that to happen, emotional regulation skill is also needed to express the emotions appropriately [30]. In view of this, our findings suggest that pet ownership can potentially decrease children’s emotional sensitivity and emotional inhibition, and lead to the development of their emotional expressiveness. For pet-owning children, human-pet interaction is one opportunity for communication. For example, since pets present a non-judgmental audience (that express unconditional love), those children might not have to feel fear of having a negative response from their emotional expression. Furthermore, even though some pets might not reciprocate children’s emotional expression, the children could still learn and develop skills of expression from interacting with their pets. Having such opportunities may encourage them to freely express their emotions, and result in the lower prevalence of poor emotional expression in later childhood.

In addition, having poor emotional expression might indicate having difficulties in recognizing their emotions or taking control of their emotions, which can be interpreted as having poor emotional regulation [31]. Deficient emotional regulation in early childhood is associated with poor development in personality or emotional function, and may lead to later mental disorders including bipolar disorder [32,33]. Our longitudinal study suggests that owning a pet might prevent emotional dysregulation in early childhood, and contribute to the development of emotional function, which is consistent with a previous study [12].

The strength of this study is that we accounted for a wide range of confounding factors in the construction of our propensity scores. Although it has been reported that pet ownership is associated with various socio-demographic factors [34,35], a majority of previous studies did not include some of the possible covariates. Matching by the propensity score also ensured that off-support inferences were avoided; children who owned pets were matched as closely as possible with children who exhibited the same probability of owning a pet (based on their background characteristics), except they did not happen to own pets. In addition, using longitudinal data to preserve the temporal sequence of exposure and outcome allowed us to determine the direction of the association between pet ownership and children’s emotional expression.

Some limitations of this study should be mentioned. First, the outcome was measured by a parental report, using a single item binary response (of yes/no). A previous study assessing emotional expression for children used 18 items [28], covering positive, negative, and inappropriate emotional expression. Thus, the assessment would be too gross to assess emotional expression skill at age 5.5 years old. Furthermore, two studies used this item and revealed the association between breastfeeding and emotional expression [26], and corporal punishment and emotional expression [27], respectively. As for parental assessment, it is reported that parents’ concerns about their child’s emotional and behavioral problems, if carefully elicited, can detect mental health problems among children aged four years or older [36]. Further study is needed to investigate the association between pet ownership and emotional expression among children using a validated scale, which use multiple items. A second limitation concerns external generalizability. In our data, the rate of pet ownership was somewhat lower than in the general population in Japan [37] as well as other countries with high rates of pet ownership, such as the U.S. (68%) and Australia (62.0%) [1,38]. Therefore, our results might not be generalizable, and further research is needed to replicate our findings in more representative studies. Third, we assessed emotional expression at 5.5 years old only, thus we did not observe the change of emotional expression skill. Although we employed propensity score matching to address bias due to the allocation of pet ownership, emotional expression skill at 3.5 years old might be higher among children with a pet. Lastly, the difference between the two groups was small, which may be due to other possible confounders that were not included in this research. For instance, unobserved children’s traits may have affected the parents’ decision to purchase a pet for their children; households with children who are more likely to be caring, prosocial and conscientious may be more likely to have pets compared to other households. These unobserved characteristics could have resulted in selection bias and residual confounding, and further research with a wider range of confounders is needed to strengthen the validity of our findings. The most rigorous test of our hypothesis would be to randomize households to pet ownership, but this may not be practically feasible due to concerns about randomizing households to keep a cat or dog in children suffering from asthma or allergy.

## 5. Conclusions

We found that pet ownership in toddlerhood was associated with better emotional expression in later childhood in Japan. We suggest that companion pets might play an important role in the development of emotional expression in childhood. Further research with a validated scale, which use multiple items to measure for the emotional expression among children, and adjusting unmeasured confounders—such as children’s temperament or emotional regulation at baseline using an instrumental variable—are needed to elucidate the mechanisms of how pet ownership can enhance the emotional development in childhood.

## Figures and Tables

**Table 1:** Prevalence (%) of pet ownership and its components (N = 31,453).

Type of pet	Boy (*n* = 16,345)	Girl (*n* = 15,108)	Total (*n* = 31,453)	*p*-Value ^a^
(1) Dog	1746 (10.7)	1557 (10.3)	3303 (10.5)	0.105
(2) Cat	697 (4.3)	679 (4.5)	1376 (4.4)	0.463
(3) Others (rabbit, small animals (such as hamster), bird, insect, fish, reptile, or others)	4883 (29.9)	3875 (25.6)	8758 (27.8)	0.004
Any type	6203 (38.0)	5120 (33.9)	11,323 (36.0)	0.004

^a^ Chi-squared test was done for examining statistical significance.

**Table 2:** Socio-demographic characteristics of children by their pet ownership condition.

Characteristics	Not-Owning A Pet (*n* = 20,130), %	Owning A Pet (*n* = 11,323), %	Total (*n* = 31,453)	*p*-Value ^a^
*Socio-demographics*				
Child’s sex				<0.001
Boy	10,142 (62.1)	6203 (38.0)	16,345	
Girl	9988 (49.6)	5120 (45.2)	15,108	
Ln (household income) ^b^, %				<0.001
First quartile	4194 (53.5)	3645 (46.5)	7839	
Second quartile	4969 (63.4)	2866 (36.6)	7835	
Third quartile	5357 (68.4)	2480 (31.6)	7837	
Fourth quartile	5610 (70.6)	2332 (29.4)	7942	
Mother’s education, %				<0.001
<**HS**	651 (53.1)	574 (48.9)	1225	
HS	10,859 (61.9)	6680 (38.1)	17,539	
Some college	5335 (67.7)	2550 (32.3)	7885	
College+	3285 (63.4)	1519 (31.6)	4804	
Father’s education, %				<0.001
<HS	1202 (55.8)	954 (44.3)	2156	
HS	9888 (61.4)	6219 (38.6)	16,107	
Some college	627 (63.0)	369 (37.1)	996	
College+	8413 (63.4)	3781 (31.0)	12,194	
Mother’s employment status				<0.001
Not-working	11,326 (68.3)	5265 (31.7)	16,591	
Full-time	3120 (61.4)	1961 (38.6)	5081	
Part-time	4171 (59.5)	2839 (40.5)	7010	
Self-employed	954 (52.9)	849 47.1)	1803	
Others	559 (57.8)	409 (42.3)	968	
Residential environment				<0.001
Predominantly residential	16,721 (66.8)	8318 (33.2)	25,039	
Retail store	719 (66.2)	367 (33.8)	1086	
Factories	276 (67.0)	136 (33.0)	412	
Rural area	2014 (47.6)	2217 (52.4)	4231	
Other	400 (58.4)	285 (41.6)	685	
No. of siblings				<0.001
0	5079 (68.9)	2288 (31.1)	7367	
1	11,477 (65.3)	6101 (34.7)	17,578	
2+	3574 (54.9)	2934 (45.1)	6508	
Living arrangement				<0.001
Not-both parents	560 (58.3)	401 (41.7)	961	
Both parents	19,570 (64.2)	10,922 (35.8)	30,492	
Poor emotional expression	4723 (23.5)	2496 (22.0)	7219 (23.0)	

^a^ Chi-squared test was done for examining statistical significance. ^b^ Annual household income, equivalized by number of people in the household.

**Table 3:** Distribution of covariates by employment condition before and after matching.

Covariate	Before Matching				After Matching				
	Not-Owning A Pet	Owning A Pet	*t*-Statistics	*p*-Values	Not-Owning A Pet	Owning A Pet	t-Statistics	% Bias Reduction	*p*-Values
Gender				<0.001					0.366
Boy, %	50.4	54.8	(reference)		55.0	54.6	(reference)		
Girl, %	49.6	45.2	−7.50		45.0	45.4	0.60	90.8	
Ln (household income) ^a^, %				<0.001					<0.001
First quartile (lowest)	20.8	32.2	(reference)		30.1	29.9	(reference)		
Second quartile	24.7	25.3	1.23		25.7	26.0	0.50	53.1	
Third quartile	26.6	21.9	−9.28		23.1	22.7	−0.56	93.2	
Fourth quartile (highest)	27.9	20.6	−14.30		21.2	21.4	−0.38	97.1	
Mother’s education, %				<0.001					0.696
<High school (HS)	3.2	5.1	(reference)		4.7	5.0	(reference)		
HS	53.9	59.0	8.67		58.8	57.9	−1.44	90.8	
Some college	26.5	22.5	−7.83		22.9	23.3	0.77	80.9	
College+	16.3	13.4	−6.88		13.6	13.9	0.53	88.9	
Father’s education, %				<0.001					0.010
<HS	6.0	8.4	(reference)		8.0	8.1	(reference)		
HS	49.1	54.9	9.90		55.0	54.1	−1.20	86.1	
Some college	3.1	3.3	0.70		3.3	3.4	0.26	55.4	
College+	41.8	33.4	−14.73		33.7	34.4	1.00	92.4	
Mother’s employment status, %				<0.001					<0.001
Not employed	56.3	46.5	(reference)		48.2	48.1	(reference)		
Full-time	15.5	17.3	4.21		17.0	17.4	0.66	81.3	
Part-time	20.7	25.1	8.91		24.6	24.3	−0.46	93.9	
Self-employed	4.7	7.5	10.12		6.8	6.7	−0.19	97.7	
Others	2.8	3.6	4.12		3.5	3.5	0.26	92.3	
Residential environment				<0.001					<0.001
Residential area	83.1	73.5	(reference)		77.3	76.1	(reference)		
Surrounded by retail shops	3.6	3.2	−1.54		2.9	3.4	1.94	−38.8	
Surrounded by plants	1.4	1.2	−1.27		1.0	1.3	1.60	−35.0	
Rural area	10.0	19.6	24.11		16.7	16.7	0.05	99.7	
Other	2.0	2.5	3.09		2.1	2.6	2.30	11.7	
No. of siblings, %				<0.001					<0.001
0	25.2	20.2	(reference)		20.0	20.9	(reference)		
1	57.0	53.9	−5.37		54.7	54.5	−0.23	95.0	
2+	17.8	25.9	17.22		25.3	24.6	−1.22	91.2	
Living arrangement, %				<0.001					0.092
Not-both parents	2.8	3.5	(reference)		3.4	3.6	(reference)		
Both parents	97.2	96.5	3.76		96.6	96.4	−0.89	71.0	

^a^ Annual household income, equivalized by number of family members.

**Table 4:** Association between pet ownership and prevalence of poor emotional expression before and after propensity score matching (*N* = 31,453).

Characteristics		Crude Model	Multivariate Model	PSM
Pet ownership (ref: either parent or none)	Owning a pet	0.94 (0.90, 0.98) *	0.94 (0.90, 0.99) *	0.94 (0.90, 0.99) *
				
*Socio-demographics*				
Gender (ref: boy)	Girl	-	0.79 (0.76, 0.83) **	-
				
Ln (household income) ^a^	Second quartile	-	1.04 (0.93, 1.15)	-
(ref: first quartile)	Third quartile	-	0.94 (0.87, 1.02)	-
	Fourth quartile	-	0.83 (0.76, 0.90) **	-
				
Mother’s education	HS	-	1.04 (0.93, 1.15)	-
(ref: <HS)	Some college	-	0.99 (0.89, 1.11)	-
	College+		0.98 (0.87, 1.11)	
Father’s education	HS	-	0.93 (0.85, 1.00)	-
(ref: <HS)	Some college	-	0.92 (0.80, 1.06)	-
	College+		0.89 (0.82, 0.97) *	
Mother’s employment status	Full-time	-	0.84 (0.79, 0.90) **	-
(ref: not employed)	Part-time	-	0.86 (0.81, 0.91) **	-
	Self-employed		0.79 (0.72, 0.87) **	
	Others	-	0.93 (0.83, 1.05)	-
Residential environment				
(ref: residential area)	Surrounded by retail stores		0.99 (0.88, 1.11)	
	Surrounded by plants		1.15 (0.98, 1.36)	
	Rural area		0.99 (0.93, 1.06)	
No. of siblings	1	-	0.94 (0.89, 0.98) *	-
(ref: 0)	2+	-	0.80 (0.75, 0.85) **	-
				
Living arrangement (ref: not living with both parents)	Both parents	-	1.10 (0.97, 1.26)	-

* *p* < 0.05, ** *p* < 0.01. PSM, propensity score matching. ^a^ Annual household income, equivalized by number of family members.

**Table 5:** Association between dog, cat, or other kinds of pet ownership, and children’s poor emotional expression.

Kinds of Pet	Crude Model	Multivariate Model	PSM
Dog	0.93 (0.85, 1.02)	0.96 (0.88, 1.05)	0.97 (0.86, 1.09)
Cat	1.06 (0.93, 1.21)	1.05 (0.92, 1.19)	1.08 (0.90, 1.28)
Other kinds of pets (rabbit, small animals (such as hamster), bird, insect, fish, reptile, or others)	0.93 (0.88, 0.99) *	0.92 (0.86, 0.97) *	0.89 (0.82, 0.95) *

* *p* < 0.05, PSM: Propensity score matching.
